# Is intuition really cooperative? Improved tests support the social heuristics hypothesis

**DOI:** 10.1371/journal.pone.0190560

**Published:** 2018-01-05

**Authors:** Ozan Isler, John Maule, Chris Starmer

**Affiliations:** 1 Centre for Decision Research and Experimental Economics, University of Nottingham, Nottingham, United Kingdom; 2 Department of Economics, Dogus University, Istanbul, Turkey; 3 Leeds University Business School, University of Leeds, Leeds, United Kingdom; 4 School of Economics, University of Nottingham, Nottingham, United Kingdom; Groupe ESC Dijon Bourgogne, FRANCE

## Abstract

Understanding human cooperation is a major scientific challenge. While cooperation is typically explained with reference to individual *preferences*, a recent cognitive process view hypothesized that cooperation is regulated by socially acquired *heuristics*. Evidence for the social heuristics hypothesis rests on experiments showing that time-pressure promotes cooperation, a result that can be interpreted as demonstrating that *intuition promotes cooperation*. This interpretation, however, is highly contested because of two potential confounds. First, in pivotal studies compliance with time-limits is low and, crucially, evidence shows intuitive cooperation only when noncompliant participants are excluded. The inconsistency of test results has led to the currently unresolved controversy regarding whether or not noncompliant subjects should be included in the analysis. Second, many studies show high levels of social dilemma misunderstanding, leading to speculation that asymmetries in understanding might explain patterns that are otherwise interpreted as intuitive cooperation. We present evidence from an experiment that employs an improved time-pressure protocol with new features designed to induce high levels of compliance and clear tests of understanding. Our study resolves the noncompliance issue, shows that misunderstanding does not confound tests of intuitive cooperation, and provides the first independent experimental evidence for intuitive cooperation in a social dilemma using time-pressure.

## Introduction

The emergence and expansion of cooperation is vital for the success of human civilisations. Early humans beat evolutionary odds, in part, by forming small groups to hunt, gather and share large mammals in the African savanna. Since then, human cooperation has evolved to support global networks of communication, production and trade [1–2]. In examples such as these where cooperation is often mutually beneficial, the tendency to cooperate is often explained via mechanisms grounded in self-interest, such as those invoking “weak reciprocity” [3–6] or fitness enhancing behaviour at the kin, group or spatial levels [7–11].

Behavioural experiments over the past fifty years, however, show that human cooperation can also be *altruistic*. For example, in social dilemma games involving anonymous and non-repeated interactions, where cooperation is neither individually beneficial nor fitness enhancing, humans across the globe exhibit significant cooperative tendencies, including a willingness to punish defectors at personal cost to themselves [1, 11–19]. Such findings have motivated a rethinking of the theoretical foundations of cooperative behaviour; in particular, it has led to an emphasis on the concept of “strong reciprocity”, understood as an evolutionarily acquired preference for cooperation, and now widely viewed as an important ingredient in explaining cooperation [1, 20–21]. However, this conception of cooperation as altruistic preference was recently challenged by the *social heuristics hypothesis* [11, 22–25] that grounds the study of cooperation in contemporary cognitive process models.

The social heuristics hypothesis draws on the well-established dual-process perspective which posits that individual decisions emerge from an interplay of two systems of thinking: A fast and frugal system that relies on heuristics and affective responses versus a slow and effortful system that relies on deliberative decision-making [26–27]. It also draws on the concept of ecological rationality, which highlights the adaptive fit between the human mind and the environment [28–30]. In particular, proponents of the social heuristics hypothesis claim that intuitive decisions reflect heuristics acquired in the course of everyday social interactions about whether or not to cooperate. Accordingly, people who have regularly benefited from cooperation in the past will tend to automatically cooperate in future social dilemmas *when acting on intuition*. However, they will be less cooperative *the more they reflect on the current situation*, as reflection enhances the salience of private costs of cooperation. In contrast, people who have not acquired such heuristics will not exhibit intuitive cooperation. Thus, a novel prediction is that any mixed population would tend to exhibit intuitive cooperation, and that the strength of this tendency would depend on the proportions of the types in the population [22–25].

This characterisation of cooperation as a psychologically-grounded social heuristic, *if correct*, has profound theoretical and practical implications across multiple disciplines including Anthropology, Biology, Economics and Psychology. The social heuristics hypothesis and its prediction of intuitive cooperation challenge not simply a more conventional view of agents as instinctively selfish [31–32] but also the more basic characterization of patterns in human cooperation as reflecting some distribution of stable and consistent prosocial attitudes [20–21]; instead, it interprets cooperation as reflecting short-cuts adapted to and norms imported from natural ecologies [23, 33–34]. Moreover, if cooperation is context-dependent then it has the potential to be manipulated–a possibility with significant policy implications. For example, policies to ‘train intuition’ in local social interactions in ways that promote intuitive cooperation may help sustain cooperation in the management of global problems such as climate change [35]. Cooperative intuitions may also be open to exploitation and hence regulation may be warranted to prevent such abuse.

Given the wide relevance of the social heuristics hypothesis, the last five years has seen a concerted effort to test the prediction of intuitive cooperation, culminating in a meta-analysis [36] and a replication project involving twenty-one labs [37]. However, two major unresolved issues have so far prevented a reliable interpretation of the accumulated data as supporting the social heuristics hypothesis. In this study, we resolve both issues and consequently provide the first strong and independent evidence for intuitive cooperation.

These unresolved issues, as we now explain, are endemic to experimental data generated via what has become a standard cognitive process manipulation protocol originally developed by Rand, Greene and Nowak for testing the social heuristics hypothesis [22]. Studies using this protocol observe behaviour in a social dilemma such as a public good game, and compare decisions made under *time-pressure* (via prompts to decide “quickly” and “in less than 10 seconds”) with those made following *forced-delay* (via prompts to think “carefully” and “for at least 10 seconds”). The time-pressure manipulation is intended to increase reliance on intuition by cutting reflective cognitive processes short. To the extent that implementations of this protocol do promote reliance on intuition, then in the absence of other confounding factors, significantly higher contributions to the public good under time-pressure compared to forced-delay may be interpreted as evidence for intuitive cooperation. However, this interpretation is seriously contested because of two potential confounds.

First and foremost, despite attempts at improvement, *noncompliance* with time-limits remains very high among time-pressured participants. In Study 6 of Rand et al [22], which introduced the protocol that we develop further in our study, 48% of time-pressured participants were noncompliant (i.e., responded after the 10 second limit). To remedy this, some subsequent studies have displayed timers on the decision screens to help participants keep track of time. Despite initial evidence from smaller-scale studies that visible timers may increase compliance [23, 38, 39], a majority (65.9%) of time-pressured participants remained noncompliant in the recent large-scale multi-lab replication project of Bouwmeester et al [37], calling into question the consistency of visible timers in achieving high rates of compliance. Currently, there is sharp disagreement in the literature about whether noncompliant participants should be excluded from the analysis. Critics of exclusion [37, 40] argue that excluding a large number of noncompliant participants voids causal identification of treatment effects (e.g., because slow responders have been shown, in response-time studies, to be less cooperative, exclusion may artificially inflate intuitive cooperation); in contrast, proponents justify exclusion by arguing that inclusion may dilute the true causal effect of intuition because noncompliant participants may have been unsuccessfully treated with the time-limit manipulations [41]. This issue is crucial because individual time-limit manipulation studies reported in the original article by Rand et al [22] as well as the large-scale meta-analysis of Rand [36] and the multi-lab replication project of Bouwmeester et al [37] provide evidence for the “intuitive cooperation” hypothesis *only* when noncompliant participants are excluded. We present a large-scale study that overcomes this problem by *familiarizing* participants with the user interface (i.e., a novel slider tool used for registering responses) and by *incentivising* compliance with time-limits, and in doing so consistently achieves comparably high levels of compliance across time-manipulation conditions.

The second unresolved issue is whether participants’ *misunderstanding* of the social dilemma situation (e.g., the public good game) confounds testing of the intuitive cooperation hypothesis. Evidence from the literature points to high levels of participant misunderstanding: Based on two questions posed after the contribution decisions have been made, 45.6% of participants in Rand et al’s [22] Study 6 and 35.9% of participants in the multi-lab replication project [37] failed to understand the public good game as a social dilemma (i.e., misconceived either the self-gain maximizing strategy, the group-gain maximizing strategy or both). It is potentially more problematic, however, if misunderstanding were particularly severe for time-pressured participants. There is some concern in the literature that this may be the case because, unlike time-pressured participants, those in the forced-delay condition have the opportunity to consolidate their understanding while deciding upon the contribution to the public good [42]. Such an asymmetric distribution of understanding across the time-limit conditions may confound tests of intuitive cooperation if misunderstanding leads participants to deviate from their own payoff maximization by mistake. However, the Rand et al protocol [22] may not provide a sound basis for testing whether there are asymmetries in understanding across time manipulation conditions because questions of understanding in this protocol are themselves *not* time-manipulated. Unlimited time for answering understanding questions may allow those participants who made contribution decisions under time-pressure to reflect on the task and in doing so to “catch-up” with forced-delayed participants in their understanding of the social dilemma situation; this may lead to an overestimation of participants’ understanding at the point in time when they made their contribution decisions.

Our study addresses the issue of understanding in two ways: First, it provides a novel method to correct for possible mismeasurement of asymmetries in understanding in the Rand et al protocol [22]. By randomly assigning time-limits to questions of understanding, we capture their direct effects on measures of understanding and test for asymmetries in understanding. Second, we compare understanding in the widely-used instructions of Rand et al (our *Short* instructions condition) to two pedagogically motivated instructional “supplements” (*Long* and *Tool* instructions conditions). While *Short* provides a test of the intuitive cooperation hypothesis in a manner comparable to the current literature [22, 37], *Long* mimics the more analytical, abstract and elaborated style of public good game instructions widely used by experimental and behavioural economists [43]; comparison of understanding in *Short* vs. *Long* is intended to check whether the comparatively brief instructions used in the recent literature on intuitive cooperation cause any unique problems of comprehension. In this comparison, researchers who rely on *Long* instructions may expect *Short* instructions to diminish understanding and to increase data noise [44], whereas users of *Short* instructions may expect *Long* to weaken time-pressure manipulation’s ability to induce intuition [22]. To address these contrasting expectations in the literature regarding *Short* and *Long*, we designed the *Tool* condition, our exploratory attempt to enable inducement of intuitive thinking via time-pressure (as expected with *Short*), while achieving high level of understanding (as expected with *Long*). Hence, we hypothesize that both *Short* and *Tool* instruction conditions would exhibit intuitive cooperation but that the *Long* conditions would not do so.

To summarize, our study is designed to resolve the noncompliance issue, to check for problems of understanding and thereby to provide improved tests of the hypothesis of intuitive cooperation. Various studies since Rand et al [22, 45], in particular those that successfully used visible timers [23, 38, 39], have simultaneously achieved high compliance and found evidence of intuitive cooperation. Nevertheless, according to the meta-analysis by Rand [36], of the 27 studies that tested the effects of time-manipulations in social dilemmas, 12 were conducted by unaffiliated groups; none of these independent studies show positive effect of time-pressure on cooperation. In an era where the “gold standard for reliability is independent replication” [46], these results have not yet been replicated by scholars unaffiliated with the original Rand et al [22] group. To the best of our knowledge, our study provides the first independent evidence of intuitive cooperation in a social dilemma using time-pressure.

## Materials and methods

### Overview

We modified the online experimental protocol reported in Study 6 of Rand et al [22] to provide enhanced evaluations of intuitive cooperation and possible asymmetries in understanding. The experiment involved a 3 (Instructions) x 2 (Cooperation decision time) x 2 (Test question decision time) between-subjects design. Having received one of three different sets of instructions (the original *short* instructions of Study 6 in Rand et al or one of two instructional supplements that include either a *long* analytical description of the task or an interactive learning *tool*) participants made contribution decisions in a one-shot linear public good game either under time-pressure or forced-delay; then they answered seven questions about the public good game, either under time-pressure or forced-delay; finally, participants completed a survey involving measures of affective states, cognitive thinking styles and numeracy. The survey questions are peripheral to the main goals of this article and they are discussed in *Supporting Information*.

### Participants

We use data from 935 participants, restricting our sample to native English speaking U.K. residents who were 18 years or older, and excluding 52 participants who failed to complete the study. Ethics approval was obtained from University of Nottingham School of Economics Research Ethics Committee, and written informed consent was obtained online from each participant at the beginning of the study. Online recruitment provided us access to a participant pool that is more representative of the general U.K. population than would be possible with laboratory experiments using university students (e.g., our sample included 24% students and 45% who were 35 years or older). Experience with the public good game has been shown to weaken intuitive cooperation [23]. We thus recruited participants via *Academic Prolific*, whose participant pool has been shown to be less experienced compared to alternatives such as *Amazon Mechanical Turk* [47]. As expected, our sample reported low levels of experience with public good experiments (14% of our participants). The sample size was chosen to achieve statistical power of 80% to detect effect sizes comparable to Study 6 in Rand et al [22]. Including a participation payment of £0.50, participants earned £1.72 on average for a study with a mean completion time of 13 minutes.

### Procedure

#### Slider training

Contributions to the public good and answers to the understanding questions were elicited via a slider tool. We modified the slider technology used in Rand et al [22] to address two potential problems. First, the slider used by Rand et al has a default position in the middle, which may have an anchoring effect on participants’ responses. Instead, our selection tool began with an empty bar with only the minimum and maximum values of the choice set visible; the slider appears only when the empty bar is clicked, and it displays the currently chosen value, which dynamically updates as the slider is moved along the bar. Second, participants in the time-manipulation studies of Rand et al [22] encounter the slider for the first time when asked to submit their contribution decisions. Forced-delay participants have time to familiarise themselves with how the slider works whereas time-pressured participants do not, and this difference may affect participants’ responses including their compliance with time-limits. To minimize differences in familiarity with the slider and increase compliance, participants in our study completed, prior to seeing the public good game instructions, a slider training screen that explained how the slider worked and then asked them to submit and verify at least one numerical value of their own choosing.

#### Public good game

We followed the experimental procedure of Study 6 of Rand et al [22], using a one-shot linear public good game with four-person groups, individual endowment of 40 pence and “marginal per capita return” of 0.5 (i.e., net individual return on units contributed to the public good). After reading instructions first on the public good game and then on the monetary consequences of noncompliance with the time limits, individuals were asked to use the slider tool to privately choose how much of their endowment to contribute to a “common project” (i.e., the public good) and how much to “keep for self”. Each penny contributed to the public good leads to a net individual loss of half a penny, while increasing total group earnings by two pennies. In terms of monetary earnings, maximisation of self-gain is achieved by keeping all the endowment, whereas group-gain maximization requires all endowments to be contributed to the public good. This constitutes a social dilemma from the individual’s perspective because self-gain and group-gain maximization cannot simultaneously be achieved.

#### Test of understanding

Having made their contribution decisions, participants were asked seven questions about the task they had just completed, with each correct answer earning them an additional 20 pence. Six of the questions assessed understanding of various aspects of the public good game, whereas one question elicited beliefs regarding other group members’ average contributions. Questions were presented on separate screens, and were of comparable length (ranging from 13 to 16 words).

The first two questions, presented in random order, jointly measure “social dilemma understanding”: *Q*_*Self*_ asks for the individual level of contribution necessary for maximizing own monetary payoff (the correct answer is no contribution) and *Q*_*Group*_ asks for the individual level of contribution necessary for maximizing monetary earnings for the whole group (the correct answer is full contribution). Following the convention in the literature [22], we categorize a participant as having understood that the public good game is a social dilemma if *both* answers are correct. The five subsequent questions were exploratory and analyses of these are presented in *Supporting Information*.

### Manipulations

#### Time-pressure vs. forced-delay

Each participant was randomly assigned to one of two time-limit conditions (time-pressure or forced-delay) *twice*, once for the public good contribution decision and then for answering the understanding questions. Based on the Rand et al [22] protocol, the instructions prompted participants to “decide quickly and choose within ten seconds” in the time-pressure condition, or to “carefully think for at least ten seconds” in the forced-delay condition. The former instructions are intended to reduce opportunities for reflective thinking thereby inducing greater reliance on intuitive processes; the latter are intended to encourage greater reliance on reflective processes.

We modified the time-manipulation protocol of Rand et al [22] to address the problems of *noncompliance* to time limits and potential *mismeasurement* of asymmetric understanding discussed in the Introduction. We addressed noncompliance by imposing pecuniary consequences for responses that lay outside the prescribed time; participants were told that noncompliant responses would be ineligible for any additional earnings from the upcoming task. This information was conveyed to participants *twice*—prior to the contribution decision screen, and prior to the questions evaluating understanding. In each case, the information was presented on a separate screen for a fixed period (15 seconds and 20 seconds respectively), intervals that were long enough for participants to clearly read the text but short enough to deny the opportunity to reflect on the upcoming task. To check whether misunderstanding is a confound we provided an independent measure of the effect of time-limits on understanding by randomly assigning participants to the time-pressure and forced-delay conditions for understanding questions; hence, any spill-over effects of the contributions task were likely to be balanced across the time-pressure and forced-delay conditions of the understanding task. We thus observe the independent effects of the time-limit manipulations on questions of understanding.

#### Public good game instructions

Each participant was randomly assigned to one of three different public good game instruction conditions (instructions are available in *Supporting Information*). For *Short*, our benchmark instructions for testing intuitive cooperation, we adopted the instructions of Study 6 in Rand et al [22] with minor modifications. To identify problems of understanding unique to *Short*, we devised two further sets of instructions, *Long* and *Tool*. These begin with the same text as *Short*, then the two examples at the end of *Short* are replaced by one or other of the two instructional supplements. *Long* mimics salient features of public good game instructions widely-used in Experimental Economics, originally designed to achieve high levels of understanding in the laboratory [15, 43]. Compared to *Short*, *Long* is not only longer (202 vs. 730 words) but is also more analytical and abstract in style, explaining the relationship between contributions and earnings via mathematical functions and examples involving numerical calculations. It is as an open question whether *Long* would improve understanding; either way *Long* may not be ideal for testing for intuitive cooperation as its analytical style may induce “reflective thinking” among *all* participants, thereby weakening the capacity of time-pressure manipulation to induce intuitive thinking [22, 40]. *Tool*, our third set of instructions, is a novel attempt to enhance understanding via self-guided experiential learning; it provides participants with an onscreen “calculator” with two inputs (own contribution level and others’ average contribution level) and simultaneously display two outputs (own earnings and others’ average earnings). Using this tool, participants are asked to verify for themselves two examples and encouraged to explore further the determination of earnings from the public good game on their own. Unlike the *Long* instructions, *Tool* conceals the functional determination of and any numerical calculations pertaining to the earnings from the public good behind the “black-box” of a calculator. We expected that, relative to *Long*, *Tool* would be less likely to induce reflective thinking; we also expected it to enhance understanding relative to *Short*. As such, the *Tool* condition is an exploratory attempt to provide an alternative to *Short* for testing intuitive cooperation.

## Results

We begin by reporting manipulation checks and data on compliance with time limits and then present our findings on intuitive cooperation and understanding. In order to provide comparability with previous research, our analysis and statistical methods follow those reported in the original work of Rand et al [22]; likewise, we report only two-tailed statistical tests throughout the article.

### Manipulation checks

Following the procedure adopted by previous research, we use the effects of time-limit prompts on response times as manipulation checks [48]. The average time taken to make contribution decisions across all three instruction conditions was 6.9 seconds with 95% confidence interval of [6.6, 7.1] for the time-pressure group and 32.9 seconds [29.9, 35.8] for the forced-delay group (n = 935; t-test on log_10_ of response time: *P* < 0.001). Similarly for understanding questions, time-pressured participants across all instructional variations spent 9.9 seconds [9.6, 10.3] on average per question, compared to 28.5 seconds [27.0, 29.9] by those under forced-delay. Thus, time-manipulation of the understanding questions motivated participants to regulate their response duration in the intended directions (n = 935; t-test on log_10_ of response time: *P* < 0.001). In short, the time-limit manipulations induced behaviourally distinct treatment groups as intended.

### Compliance with time limits

**Fig 1:**
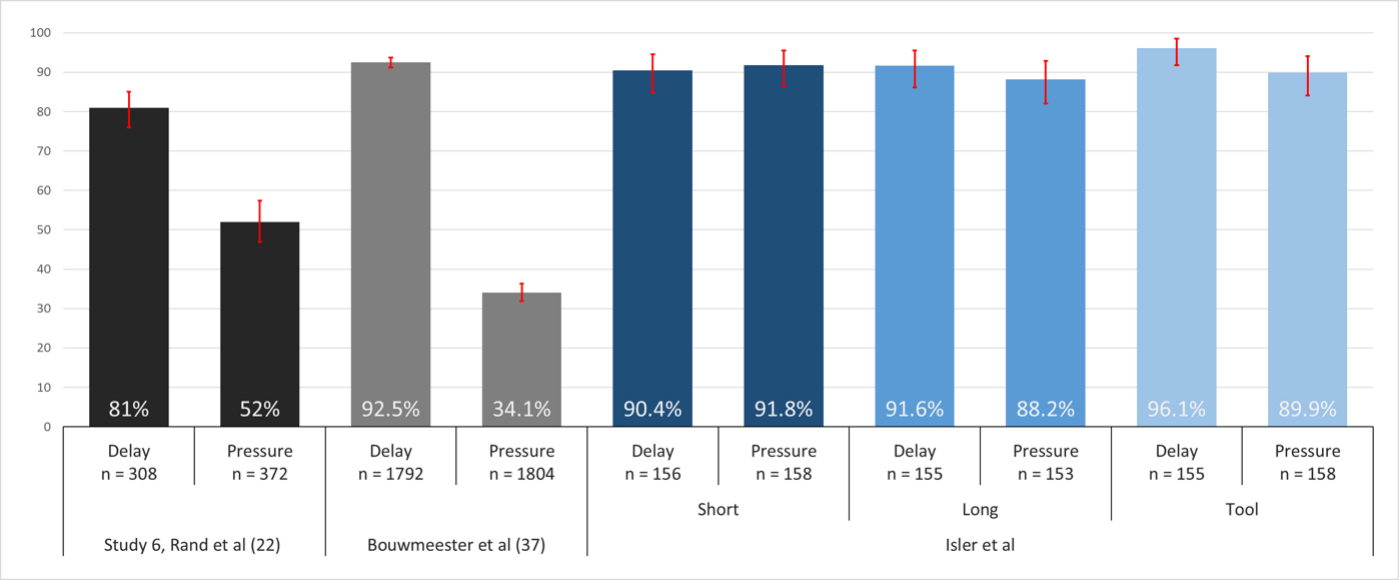
Time-limit compliance rates for forced-delay and time-pressure conditions. Error bars denote 95% confidence intervals.

As illustrated in Fig 1, our modified protocol achieved high compliance in both time-limit conditions of the public good game consistently across all instructional variations. The four monochrome bars in the figure represent the time-limit compliance rates found in Study 6 of Rand et al [22] and Bouwmeester et al [37], showing significantly lower compliance under time-pressure as compared with forced-delay. The six blue bars represent our study, showing compliance rates that ranged from 88.2% to 96.1%. Overall, compliance in our study was 90.0 [86.9, 92.5] in time-pressure vs. 92.7% [90.0, 94.9] in forced-delay conditions of the public good game, a statistically insignificant difference (n = 935; χ^2^ test: *P* = 0.139). Thus, our modified time-manipulation protocol resolved the crucial issue of low and asymmetric compliance rates for the public good game and improved the likelihood of correspondence between alternative tests of intuitive cooperation (i.e., inclusion vs. exclusion of noncompliant participants).

Compliance to time-limits imposed on the two understanding questions varied considerably: Compliance was 78.3% [74.3, 82.0] in time-pressure vs. 83.7% [80.1, 87.0] in forced-delay conditions for *Q*_*Group*_ (χ^2^ test: *P* = 0.033), and was 61.6% [57.0, 66.0] in time-pressure vs. 88.1% [84.8, 90.9] in forced-delay conditions for *Q*_*Self*_ (n = 935; χ^2^ test: *P* < 0.001). Given that the same sample of participants was largely compliant when making contributions to the public good, the difference in compliance rates under time-pressure between *Q*_*Group*_ and *Q*_*Self*_ probably reflects a previously unnoticed difference in the difficulty of answering *Q*_*Self*_ relative to *Q*_*Group*_. We present more evidence supporting this novel finding in *Supporting Information* as it does not pertain to this study’s primary motivation.

### Intuitive cooperation

**Fig 2:**
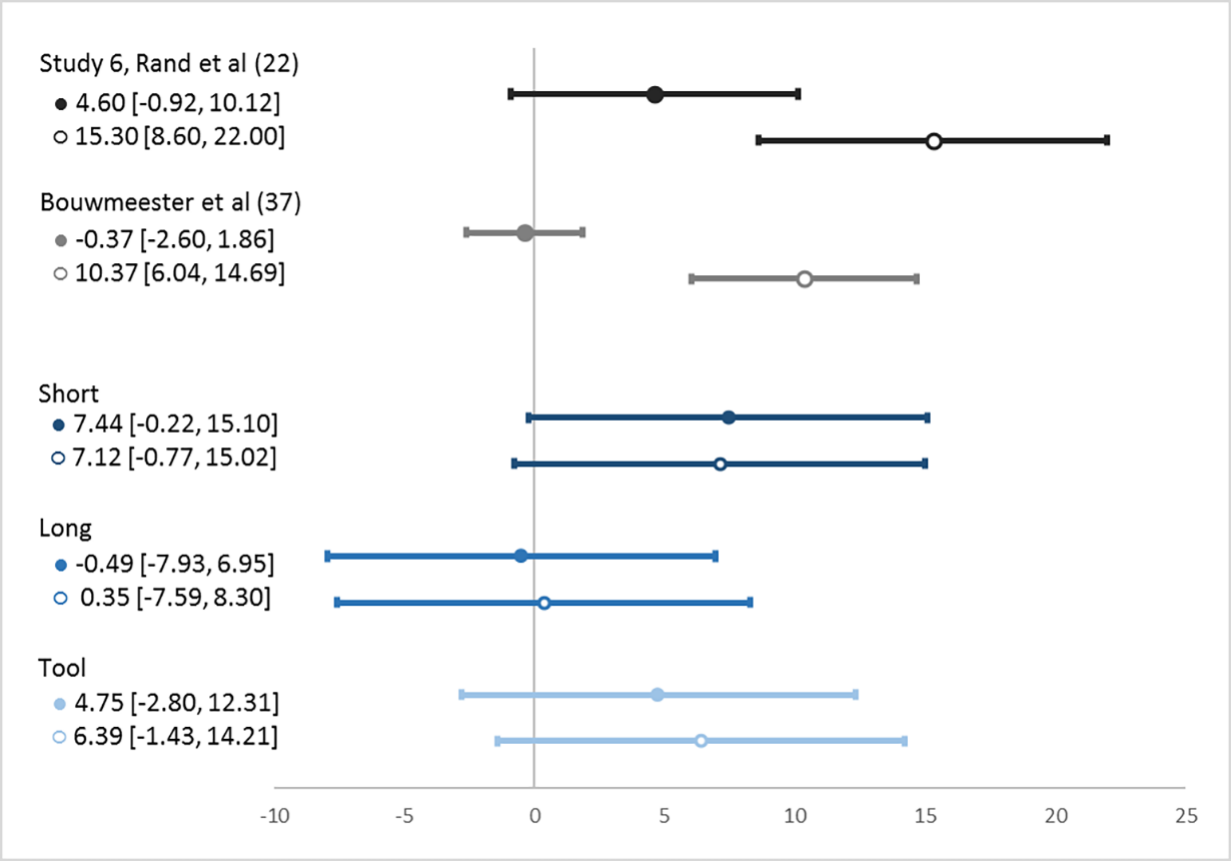
Time-pressure minus forced-delay percentage point difference in contributions. Circles denote average contributions. Filled circles (●) include and empty circles (○) exclude noncompliant participants. Lines crossing the circles denote 95% confidence intervals.

Fig 2 depicts treatment effect sizes as percentage point differences in contributions between the time-pressure and forced-delay conditions. The four monochrome lines at the top summarize the findings of Study 6 in Rand et al [22] and the multi-lab replication project of Bouwmeester et al [37], where the lack of overlap between the two confidence intervals within each study demonstrates that alternative analyses of the data (i.e., inclusion vs. exclusion of noncompliant participants) provide incongruent results for studies with low rates of compliance. The six blue lines at the bottom represent our study. Notice that, for each instructional variation in our study (i.e., *Short*, *Long* and *Tool*), very similar confidence intervals emerge regardless of whether noncompliant participants are included or excluded from the analysis.

We now test for evidence of intuitive cooperation focusing first on *Short*, our attempt to replicate Study 6 in Rand et al [22]. Using all observations in *Short*, including participants who did not comply with the time-limits, we find a difference of 7.44 [-0.22, 15.10] percentage points between time-pressure and forced-delay conditions. This positive effect of time-pressure on cooperation is statistically significant (n = 314; ranksum, *P* = 0.025). As expected from the high and symmetric levels of compliance to time-limits in our public good game, these results also hold when we exclude noncompliant participants from the analysis: In this case, the difference between the two conditions is 7.12 [-0.77, 15.02] percentage points, and the positive effect of time-pressure on contributions to the public good remains statistically significant (n = 286; ranksum, *P* = 0.039). We thus provide the first independent evidence of intuitive cooperation using a time-manipulation protocol that is free from complications of noncompliance.

As previously discussed, the analytical style of *Long* may weaken the inducement of intuition. In line with this expectation, the percentage point difference in contributions between all time-pressured and forced-delay participants in *Long* is -0.49 [-7.93, 6.95], which is not statistically distinguishable from zero (n = 308; ranksum, *P* = 0.741). The *Tool*, on the other hand, was expected to exhibit intuitive cooperation by minimizing overall inducement of reflective thinking while enhancing understanding as compared with *Short*. Although the difference in contributions between the two time-limit conditions is not statistically significant (n = 313; ranksum, *P* = 0.380), contributions in *Tool* among all participants under time-pressure are nevertheless 4.75 [-2.81, 12.32] percentage points higher than those under forced-delay. The fact that the bulk of the 95% confidence intervals for *Tool* lay to the right of zero is consistent with the interpretation that *Tool* exhibits intuitive cooperation and that our study was underpowered to detect its smaller effect. Finally, we pool together all observations from *Short* and *Tool*, the two instruction conditions expected to exhibit intuitive cooperation; we observe that contributions are 6.09 [0.70, 11.48] percentage points higher under time-pressure than under forced-delay and we find this effect to be statistically significant (n = 627; ranksum, *P* = 0.033).

### Understanding of the social dilemma

We first estimate the extent of social dilemma misconception in *Short* and compare it to the two instructional supplements. As described in the Methods section, we measure social dilemma understanding (i.e., conception of the public good game as a social dilemma) with a variable that equals 1 if *both* test questions that constitute the social dilemma (*Q*_*Self*_ & *Q*_*Group*_) are answered correctly, and zero otherwise. Only 43.3% [37.8, 49.0] of participants in the *Short* instructions condition exhibited correct social dilemma understanding, raising the question whether similarly high levels of misunderstanding observed in the literature are due to the briefness of the instructions provided by Rand et al [22]. Surprisingly, despite extended analytical descriptions and numerical examples, social dilemma understanding in *Long* (31.8% [26.7, 37.3]) was 11.5 percentage points *lower* than *Short* (n = 622; χ^2^ test: *P* = 0.003). Comparing *Short* to *Long*, the high levels of misunderstanding found in the literature do not seem to stem from the briefness of instructions used; in contrast, the analytical, abstract and elaborated instructional tradition of Economics leads to poorer comprehension, at least in our online environment. On the other hand, understanding in *Tool* (51.4% [45.8, 57.1]) was 8.1 percentage points higher than *Short* (n = 627; χ^2^ test: *P* = 0.042). Comparing *Short* to *Tool*, we see that enhanced opportunities for learning via self-guided examples are effective in improving understanding of the decision problem as a social dilemma.

**Fig 3:**
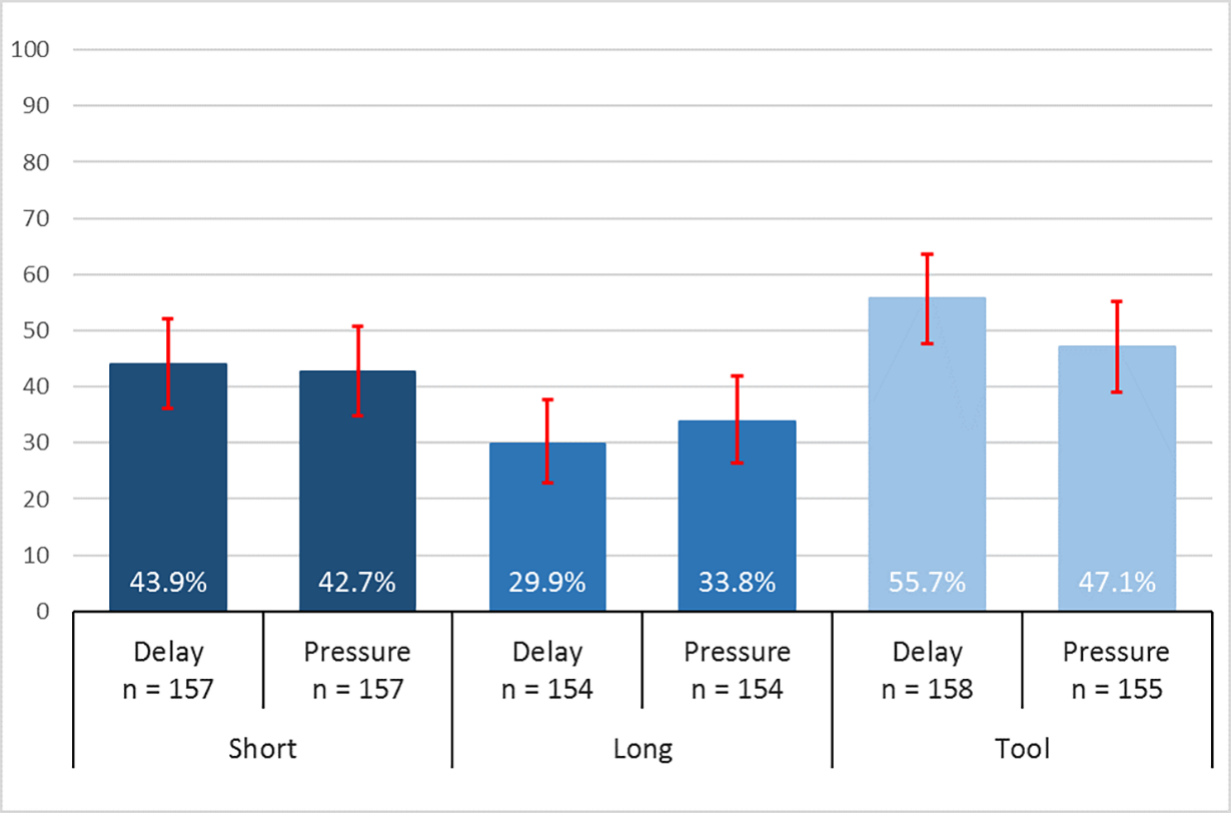
Rates of social dilemma understanding for forced-delay and time-pressure conditions imposed on the understanding questions. Error bars denote 95% confidence intervals.

Despite these high levels of misunderstanding and in line with findings in the literature, we do not find the public good game contributions to depend on social dilemma understanding either in *Short*, *Long* or *Tool* conditions (Ranksum: *P* > 0.250 for each condition). To provide a more precise test whether misunderstanding may be confounding our finding of intuitive cooperation, we now check for asymmetries in understanding across the time-limit manipulations of the understanding questions. Fig 3 depicts rates of social dilemma understanding for each instructional variation based on whether the questions were answered under time-pressure or forced-delay. Even with the enhanced measure of asymmetries, we observe no statistical difference in social dilemma understanding between time-pressured and forced-delayed test question groups for *Short*, *Long* or *Tool* conditions (χ^2^ test: P = 0.820 for *Short*; P = 0.463 for *Long*; P = 0.128 for *Tool*). Hence, our study finds no support for the suggestion that measures of understanding in the literature are biased by asymmetries between the time-pressure and forced-delay conditions.

Finally, as summarized in Table 1, we ran a set of tobit regressions with model specifications similar to Study 6 of Rand et al [22], and replicate their finding that intuitive cooperation continues to hold when controlling for understanding (R1 vs. R2). As an additional analysis, we consider the interaction between understanding and time-limit conditions applied during the contribution decisions, and find that the effect of intuitive cooperation exists *only* for those who have the correct social dilemma understanding (R3). This finding is in line with the empirical results of Strømland, Tjøtta & Torsvik [42] as well as with the predictions of the evolutionary game theoretic model of Bear & Rand [24], where reflection diminishes cooperation only if people are aware that the decision context is a social dilemma. This latter result indicates that the presence of a large percentage of participants with misconceptions may undermine the effect size of intuitive cooperation. Overall, these results suggest that misunderstanding does not confound our finding of intuitive cooperation in *Short*.

**Table 1:** Tobit regressions of contributions made (%) in *Short* instructions condition.

Model	Predictor	*b*	*SE*^*a*^	95% CI	*p*
R1	Time-limits (Pressure = 1, Delay = 0)	19.32	8.16	[3.25, 35.37]	0.02
R2	Time-limits (Pressure = 1, Delay = 0)	19.03	8.11	[3.06, 35.00]	0.02
	Understanding (Correct = 1, Incorrect = 0)	-3.98	8.41	[-20.53, 12.57]	0.64
R3	Understanding Incorrect (Pressure = 1, Delay = 0)	11.67	9.63	[-7.28, 30.63]	0.23
	Understanding Correct (Pressure = 1, Delay = 0)	28.97	13.77	[1.88, 56.06]	0.04

Regressions control for age and gender.

^a^ Denotes robust standard errors.

## Discussion

The social heuristics hypothesis predicts a tendency for intuition to be cooperative. However, two potential confounds have until now inhibited a clear interpretation of the accumulated evidence. The goal of this study was to eliminate these confounds and thereby to conduct stronger tests of the prediction of intuitive cooperation. Our modified experimental protocol succeeded in achieving this goal, and concurrently provided the first independent replication of the phenomenon of intuitive cooperation in a social dilemma using time-pressure.

Our resolution of the issues of compliance and understanding relied on modifications to the original protocol. To increase compliance, we allowed familiarization with the user interface and introduced pecuniary incentives; high rates of compliance across the treatment conditions generated congruent tests of the hypothesis of intuitive cooperation and hence avoided the polarising issue of whether to exclude noncompliant participants from the analysis. To check whether misunderstanding confounds these hypothesis tests, we introduced time-limits on understanding questions, and we compared the relatively brief instructions used in the original protocol with pedagogical alternatives; we found no evidence for confounds.

Overall, our findings allow us to view competing interpretations of the research on intuitive cooperation in new light. In particular, three pivotal studies in the literature provide evidence for the intuitive cooperation hypothesis *only* when noncompliant participants are excluded [22, 36, 37]. While Bouwmeester et al [37] argue that exclusion may bias the results because slow responders are found to be less cooperative, Rand [41] justifies exclusion by arguing that noncompliance may indicate a failure to induce intuitive thinking. Our successful minimization of noncompliance, combined with our finding of intuitive cooperation, not only supports Rand’s claim that noncompliance to time-limits may indicate manipulation failure but also redeems time-pressure as a viable method for inducing intuition. In sum, our study avoids the key problems of misunderstanding and noncompliance that have confounded interpretation of the prior evidence, and provides new and stronger support for the claim that cooperation is *indeed* intuitive.

The strong evidence we find for the social heuristics hypothesis has important implications for the study of cooperation. First, our finding supporting intuitive cooperation implies that cooperation in anonymous and non-repeated social dilemma experiments are in part due to weak reciprocators who decide intuitively, and who may have inflated previous measures of strong reciprocity in studies that did not distinguish between the dual-processes. Nevertheless, participants in our study contributed more than half of their endowment to the public good even when deciding reflectively. It remains to be seen whether this is evidence of strong reciprocity as is often argued or evidence of heuristics that are resistant to change. Second, our findings are in line with the argument that dual cognitive processes play a significant role in the relationship between learning and human cooperation [22–25, 34, 35, 38, 39]. In this relationship, the role of culture in general and social learning in particular remain understudied. The social heuristics hypothesis predicts that people from backgrounds with higher frequencies of mutually beneficial social interactions would exhibit stronger intuitive cooperation. Given our findings, a more stringent test of the social heuristics hypothesis could be achieved by investigating predictions of intercultural differences in intuitive cooperation [33, 34]. Finally, the phenomenon of intuitive cooperation suggests new avenues for policy-making. As evidenced by growing global common resource problems such as climate change and antimicrobial resistance, strong reciprocity in the relevant populations may not be sufficient to achieve the levels of cooperation necessary for their resolution. At the same time, cooperative experiences in the lab have been shown to have positive spill-over effects in social dilemmas [35], and intuitive cooperation has been shown to be present in everyday interactions [49]. Hence, policy goals may be fostered by exposing citizens to mutually beneficial local cooperative experiences in order to harness intuitive cooperation spill-overs in the context of global social dilemmas [50].

## Supporting information

S1 File (DOCX)

S1 Dataset (XLS)
