# Supplementary material for: Is intuition really cooperative? Improved tests support the social heuristics hypothesis
Source: PLoS One. 2018 Jan 5;13(1):e0190560. doi: 10.1371/journal.pone.0190560 (PMC5755815; doi:10.1371/journal.pone.0190560)
Supplement: S1 File — (DOCX) [file pone.0190560.s001.docx]

**Supporting** **Information:**

**Is intuition really cooperative?**

**Improved tests support the social heuristics hypothesis**

Ozan Isler, John Maule, Chris Starmer

1. **Questions of Understanding**

**As described in the article, understanding is measured in the literature with a variable constructed from answers to two questions (Q_self_ & Q_group)_. These two standard questions as well as the five other exploratory questions are listed in Table A. For all but one question, the second column of the table reports the percentage of correct answers for the whole sample; for Q_belief_ the reported statistic is the average answer. A striking feature of the responses, particularly pertinent to the article, is that when we consider the two standard questions separately, performance on Q_self_ is significantly lower than Q_group_ (**n = 935; **McNemar,** *P* < 0.01). Performance on **Q_group_** was the highest among all questions (80%), while performance on **Q_self_ was only 46%. A similar pattern is found in Bouwmeester et al [1], where the percentage of correct answers** was 74.3% **for Q_group_ and 60.4% for Q_self_. While these results suggest an interesting avenue for further investigation, the fact that people appear to be significantly better at understanding the action required for group-gain maximizing is arguably in line with the hypothesis of intuitive cooperation (also see Numeracy below).**

**Table A.** **Performance on Questions of Understanding (% Correct^a^)**

| Q_self_ | 45.7 | “What level of contribution from you earns the highest payoff for you personally?” |
| --- | --- | --- |
| Q_group_ | 80.2 | “What level of contribution from you earns the highest payoff for the group as a whole?” |
| Q_belief_ | 56.8 | “What will be the average of the contributions to the project from others in your group?” |
| Q_equal_ | 70.5 | 1. “If others contribute 20 p each, what contribution from you leads to equal payoffs for all? |
|  |  | 2. “If others contribute 10 p each, what contribution from you leads to equal payoffs for all? |
|  |  | 3. “If others contribute 30 p each, what contribution from you leads to equal payoffs for all? |
| Q_calculation_ | 14.2 | 1. “If you contribute 20 p while others contribute 0 p, what would be your payoff?” |
|  |  | 2. “If you contribute 0 p while others contribute 60 p in total, what would be your payoff?” |
|  |  | 3. “If everyone, including you, contributes 20 p each, what would be your payoff?” |
| Q_time-limit_ | 66.4 | “What will be your payoff from this question if you answer in more than 10 seconds?” |
| Q_members_ | 66.8 | “Including you, how many people are assigned to your group for the interaction?” |

The first two questions question were randomly presented; the last five questions were presented in the order displayed. For Q_equal_ and Q_calculation_, participants were randomly presented one of the three alternatives.

^a^ Average of answers reported for Q_belief_ (%).

1. **Numeracy**

The Berlin Numeracy Test captures an individual’s ability to understand and use numbers, basic logic and quantitative reasoning skills; those higher in numeracy deliberate more when making decisions. We used the adaptive version of the test as the last activity of the experimental session [2]. Numeracy (measured as a discreet score between 1 and 4) was strongly associated with task understanding; those scoring higher on the numeracy test were more likely to answer both questions correctly (n = 887; χ^2^ test: *P* < 0.001). In particular, among those who were in the lower half of the numeracy scale (i.e., those who scored 1 or 2; n = 598) **the percentage of correct answers** was 73.6% **for Q_group_ and 36.1% for Q_self_, whereas among those who scored 3 or 4 on the numeracy scale (n = 289), the percentage of correct answers** was 93.1% **for Q_group_ and 61.9% for Q_self_. In short, participants found it easier to correctly answer Q_group_ than Q_self_** regardless of numeracy. Nevertheless, numeracy did not have a statistically significant effect on contributions (n = 887; ranksum, *P* = 0.811), giving further support that misunderstanding does not confound our tests of intuitive cooperation.

1. **Affect**

The changes in affect that occur when participants are put under time pressure [3] have yet to be investigated in the Rand et al protocol [4]. To explore such changes we measured participants’ affective states following completion of the understanding questions, presenting them with 15 affect terms taken from PANAS [5] and those used for assessing the impact of time pressure by Maule et al [3]. Participants indicated the extent to which they felt the state described using a five-point scale (from “not at all” to “extremely”). Factor analysis revealed three factors with items loading above 0.4 used to determine a name for each (see Table B) and to develop a scale for measuring each factor (score based on equal weighting of these items).

**Table B. Solution to Factor Analysis**

| **Stress** | **Sadness** | **Positive Affect** |
| --- | --- | --- |
| "Pressured" | "Afraid" | "Confident" |
| "Tense" | "Depressed" | "Energetic" |
| "Calm"^a^ | "Tired" | "Elated" |
| "Stressed" | "Irritable" | "Alert" |
| "Confident"^a^ |  | "Interested" |
| "Afraid" |  |  |

“Guilty” and “surprise” are excluded due to low communality scores (< 0.3).

^a^ Denotes items with negative loading.

Analysis of variance revealed significantly higher levels of *Stress* for participants under time-pressure as compared with forced-delay while deciding how much to contribute (2.77 vs 2.57 respectively, *P* < 0.001) and while answering the understanding questions (2.85 vs 2.50 respectively, *P* < 0.001). The analysis also revealed higher levels of *Sadness* under time-pressure as compared to forced-delay during the contributions phase (1.64 vs 1.55 respectively, *P* = 0.033) and higher levels of *Positive Affect* for those under time-pressure during the understanding question phase (2.74 vs 2.59 respectively, *P* = 0.002). Finally, guilt (excluded from the main analysis) is negatively correlated with contributions (r = -0.123, *P* < 0.001), indicating that those who contributed less felt more guilty. These findings show that the Rand et al protocol [4] induces changes in affect, some of which are quite persistent – time-pressure induced increases in *Stress* and *Sadness* at the contribution phase are detectable later, after participants completed the understanding question phase. Since changes in affect are known to influence thinking and decision making [6] further research is needed to clarify the impact of these changes on contributions to the public good and the intuitive cooperation hypothesis.

1. **Instructions**

“Slider Training” screens are shown prior to the public good game instructions. The text in black under “Public Good Game Instructions for *Short*” is also part of the instructions for *Long* and *Tool* conditions. The text in red denotes part of the *Short* instructions that is replaced by either the *Long* or the *Tool* “Instruction Supplements” provided below. “Compliance Incentivization” and “Decision” screens are used in all instruction conditions and vary only across the time-pressure and forced-delay conditions.

*Short* instructions are based on Study 6 of Rand et al [4] with minor modifications: We deleted “(in increments of 2 units: 0, 2, 4, 6 etc.)” as contributions could be made in increments of one; we excluded two seemingly “loaded” phrases (“everyone’s money will double” and “Thus you personally lose money on contributing”); we deleted “Neither you nor the other people receive any bonus other than what comes out of this interaction” as understanding questions allowed additional earnings; we replaced “cents” with “pence”.

**Slider Training Screens**

**First Screen**


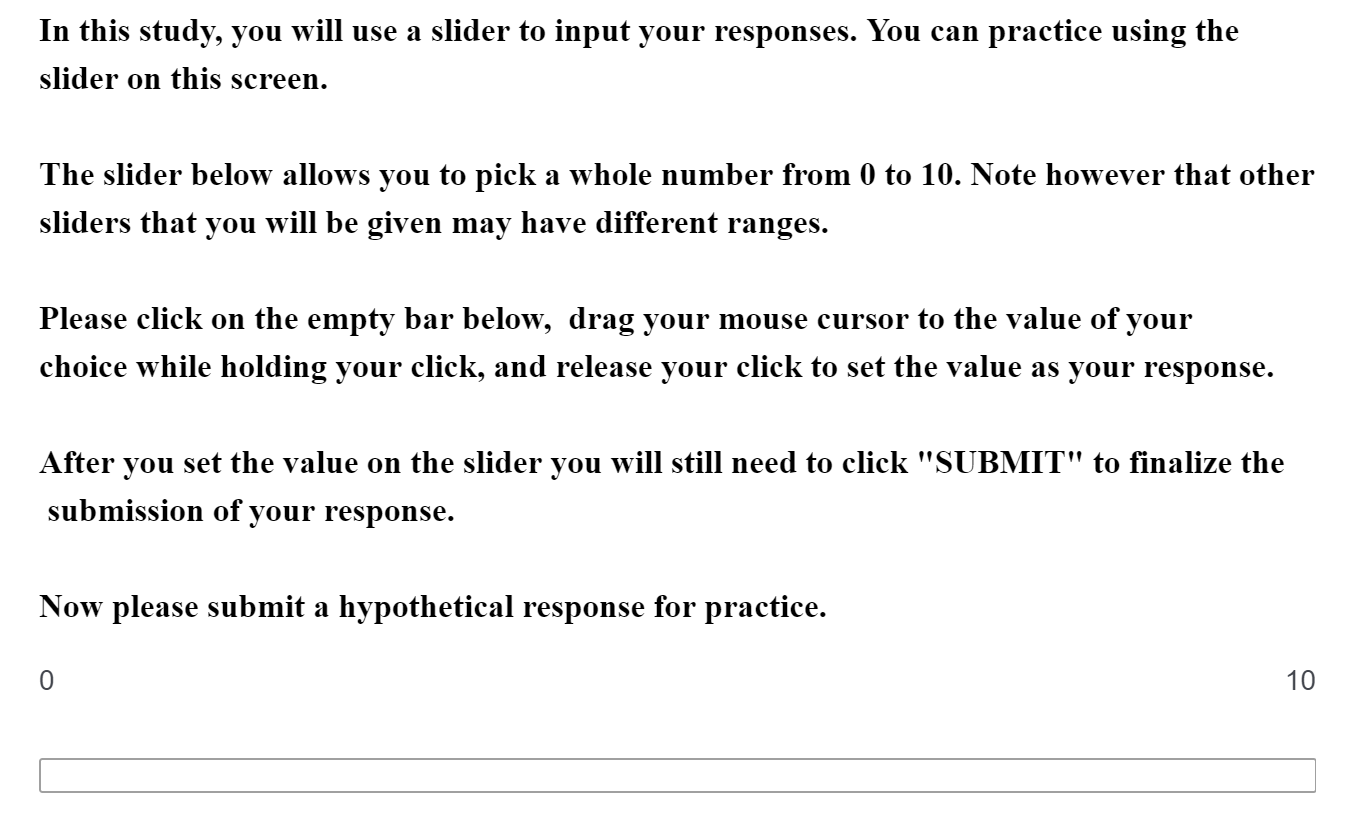


**Second Screen**


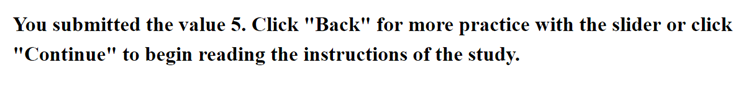


**Public Good Game Instructions for *Short***

**You have been randomly assigned to interact with 3 other people. All of you receive this same set of instructions. You cannot participate in this study more than once.** 

**Each person in your group is given 40 pence for this interaction (in addition to the 50 pence you received already for participating).**

**You each decide how much of your 40 pence to keep for yourself, and how much (if any) to contribute to the group’s common project.**

**All money contributed to the common project is doubled, and then split evenly among the 4 group members.**

**Thus, for every 2 pence contributed to the common project, each group member receives 1 penny.**

**If everyone contributes all of their 40 pence, each of you will earn 80 pence.**

**But if everyone else contributes their 40 pence, while you keep your 40 pence, you will earn 100 pence, while the others will earn only 60 pence. That is because for every 2 pence you contribute, you get only 1 penny back.**

**The other people are REAL and will really make a decision – there is no deception in this study.

Once you and the other people have chosen how much to contribute, the interaction is over.**

**Instruction Supplement for *Long***

**[…] We will now describe how earnings from the interaction are calculated in more detail.**

***

**The money you will earn from the interaction will be equal to the sum of two parts that we call “amount you kept” and “earnings from the common project”:**

**Earnings from the interaction = Amount you kept + Earnings from the common project**

**Your earnings from the interaction will depend on the amount you keep for yourself and the amounts contributed to the common project.**

*******

**Your earnings from “amount you kept” will depend only on the amount you contribute to the common project:**

**Amount you kept = 40 - Amount you contribute to the common project**

**Thus, for every 2 pence you contribute to the common project, you would earn 2 pence less from amount you kept.**

*******

**Your “earnings from the common project” will depend on the sum of all amounts contributed to the common project by all four people in the interaction:**

**Earnings from the common project = 0.5 x (Sum of all amounts contributed to the common project)**

**Thus, for every 2 pence you contribute to the common project, each group member, including you, receives 1 penny from the project. Similarly, you receive 1 penny from the common project for every 2 pence contributed to the project by one of the other three people in your group.**

*******

**Therefore, “earnings from the interaction” will be equal to:**

**Earnings from the interaction = (40 - Amount you contribute to the common project) + (0.5 x Sum of all amounts contributed to the common project)**

**We will now go through a series of examples to illustrate how earnings from the interaction are calculated.**

*******

**Suppose everyone keeps all of their 40 pence for themselves.**

**Then, each person would have 40 - 0 = 40 pence from amount kept. The sum of all amounts contributed to the common project would be (0 + 0 + 0 + 0) = 0 pence, and thus each person would earn 0.5 x 0 = 0 pence from the project.**

**Earnings from the interaction for each person thus equal (40 - 0) + (0.5 x 0) = 40 pence.**

*******

**Suppose everyone contributes all of their 40 pence to the common project.**

**Then, each person would have 40 - 40 = 0 pence from amount kept. The sum of all amounts contributed to the common project would be (40 + 40 + 40 + 40) = 160 pence, and thus each person would earn 0.5 x 160 = 80 pence from the project.**

**Earnings from the interaction for each person thus equal (40 - 40) + (0.5 x 160) = 80 pence.**

*******

**Suppose everyone else contributes all of their 40 pence to the common project, while you keep your 40 pence.**

**Since you contributed 0 pence to the common project, your earn 40 - 0 = 40 pence from amount you kept. Since each of the other three people contributed 40 pence to the common project, they each earn 40 - 40 = 0 pence from amount kept.**

**The sum of all amounts contributed to the common project is (0 + 40 + 40 + 40) = 120 pence, and so everyone will have 0.5 x 120 = 60 pence each from the project.**

**Your earnings from the interaction thus equal (40 - 0) + (0.5 x 120) = 100 pence. Earnings of each other person from the interaction thus equal (40 - 40) + (0.5 x 120) = 60 pence.**

**Instruction Supplement for *Tool***

**[…]** We will now describe how earnings from the interaction are determined in more detail.

***

The tool below demonstrates the relationship between contributions to the common project and earnings from the interaction. ​Under the "Inputs" section on the left, the top slider allows you to vary your hypothetical level of contribution and the bottom slider allows you to vary the hypothetical average level of contributions from the other three people in your group (in pence). The "Outputs" section on the right specifies what "your earnings" and what "others' average earnings" from the interaction would be if contributions were as stated in the "Inputs" section. 

By default, both your contribution and other's average contribution to the common project are set to zero. Hence, the default settings of the tool demonstrate that if everyone keeps all of their 40 pence for themselves, then earnings from the interaction for each person would be 40 pence. 

Using the tool, please verify that earnings from the interaction would be as stated in each of the examples below:

1) Suppose everyone contributes all of their 40 pence to the common project.

Then earnings from the interaction for each person would be 80 pence.

2) Suppose everyone else contributes all of their 40 pence to the common project, while you keep your 40 pence.

Then your earnings from the interaction would be 100 pence and others would earn 60 pence each.


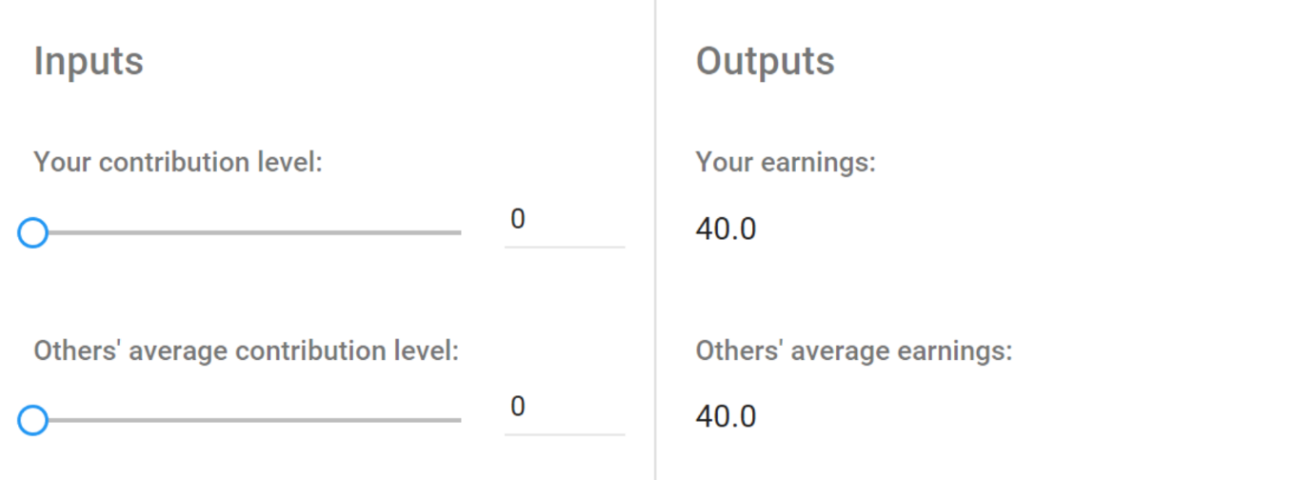


Now please use the tool to explore other examples. Familiarizing yourself with the relationship between contributions to the project and earnings from the interaction should help you make your decision.

**Compliance Incentivization Screen: Public Good Contributions (fixed period of 15 seconds)**

**Time-Pressure**


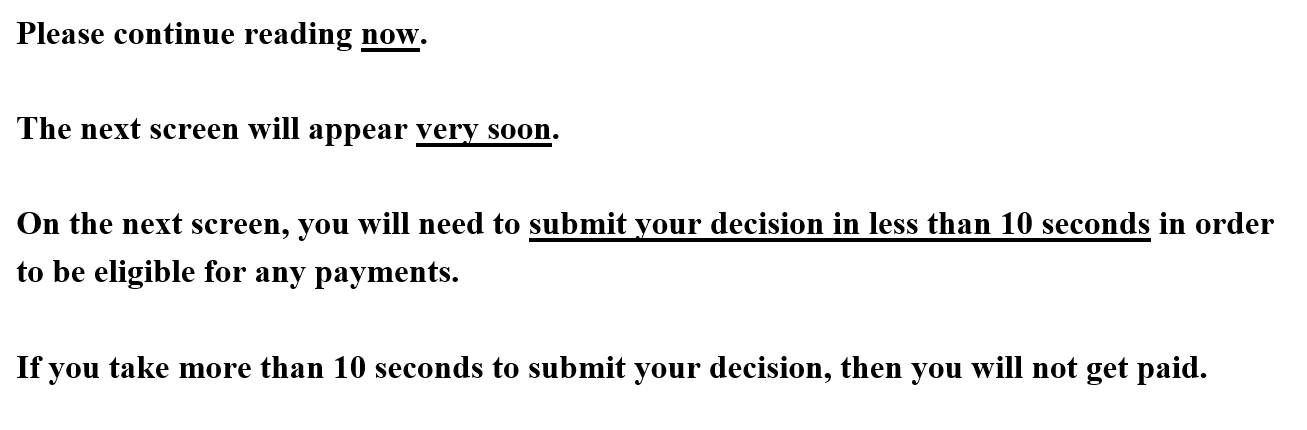


**Forced-Delay**


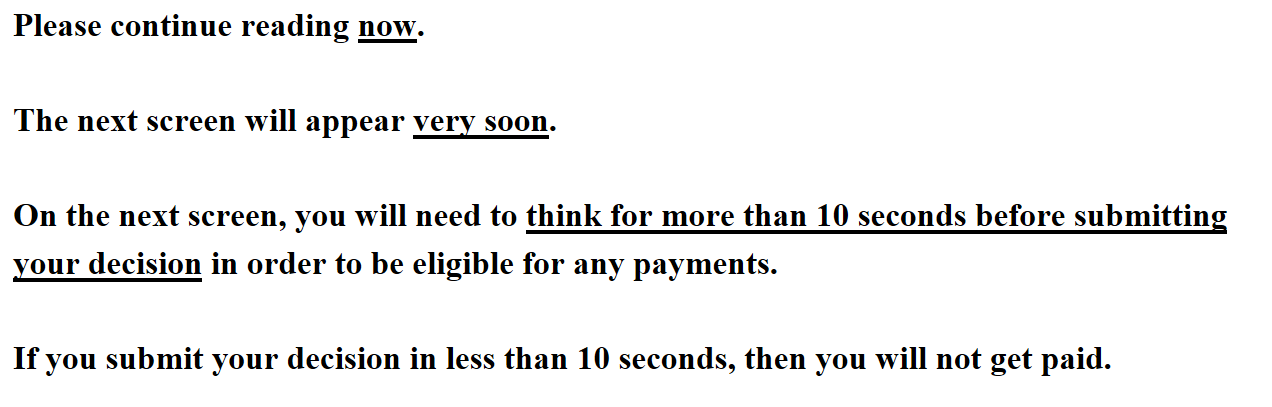


**Decision Screen: Public Good Contributions**

**Time-Pressure**


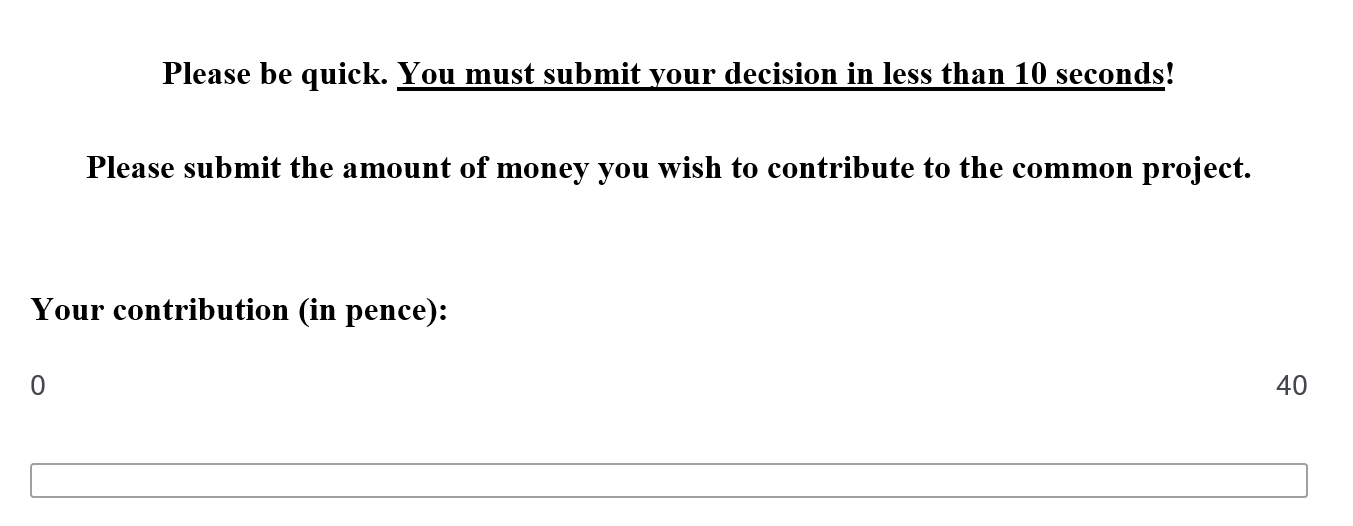


**Forced-Delay**


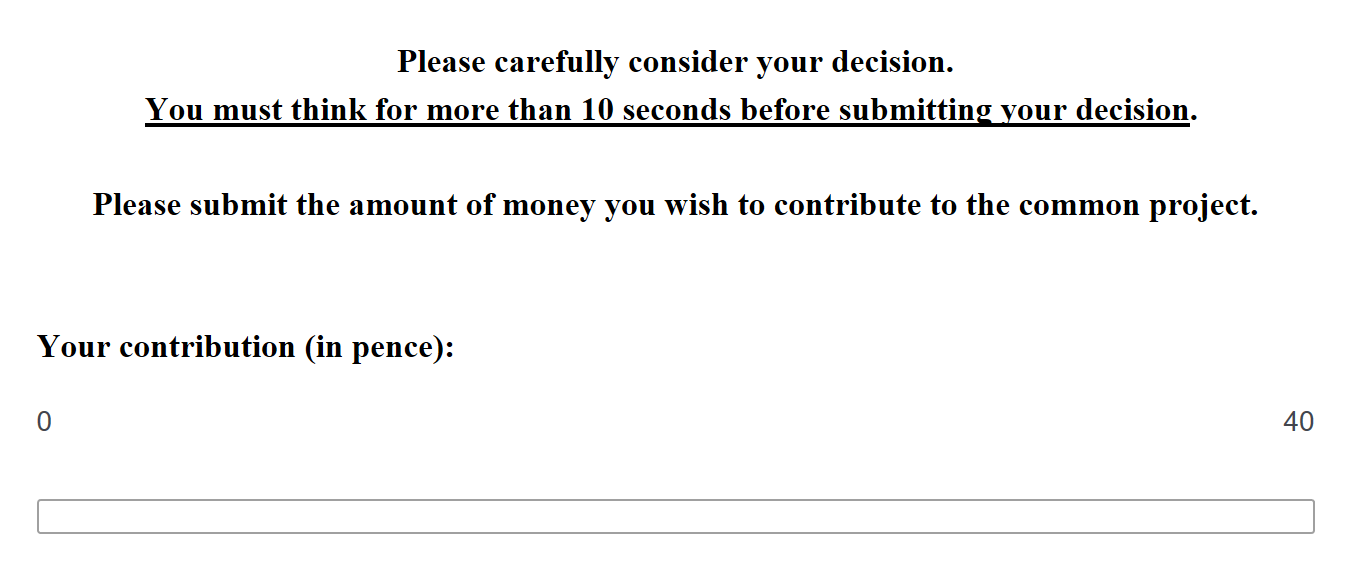


**Compliance Incentivization Screen: Understanding Questions (fixed period of 20 seconds)**

**Time-Pressure**


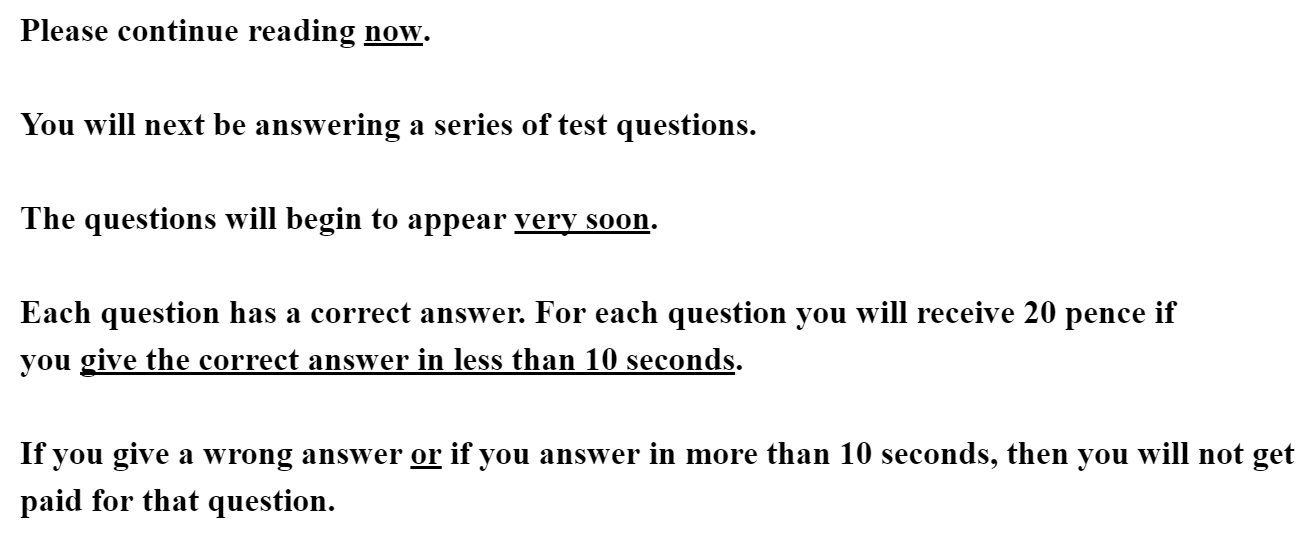


**Forced-Delay**


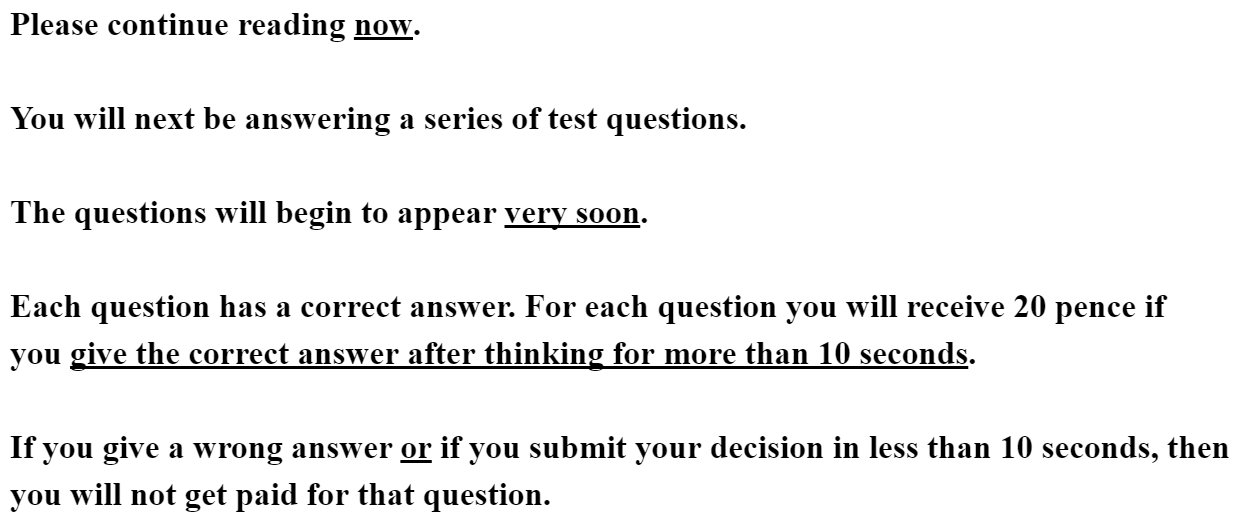


**Decision Screen: Understanding Questions**

**Time-Pressure**


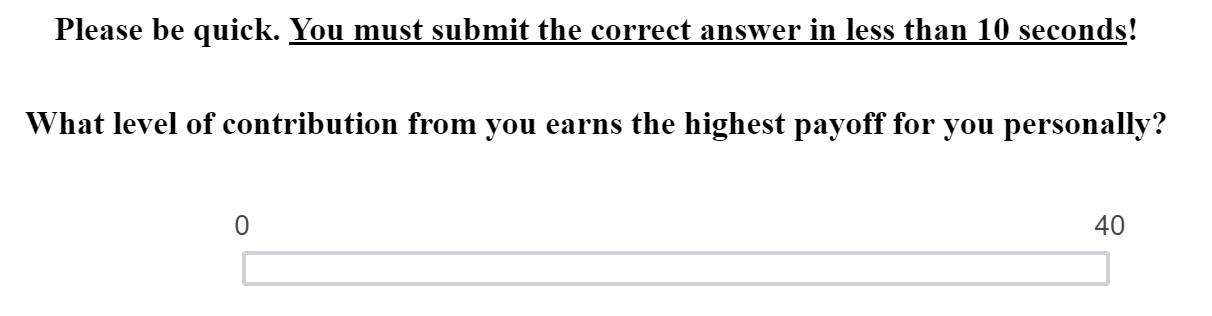


**Forced-Delay**


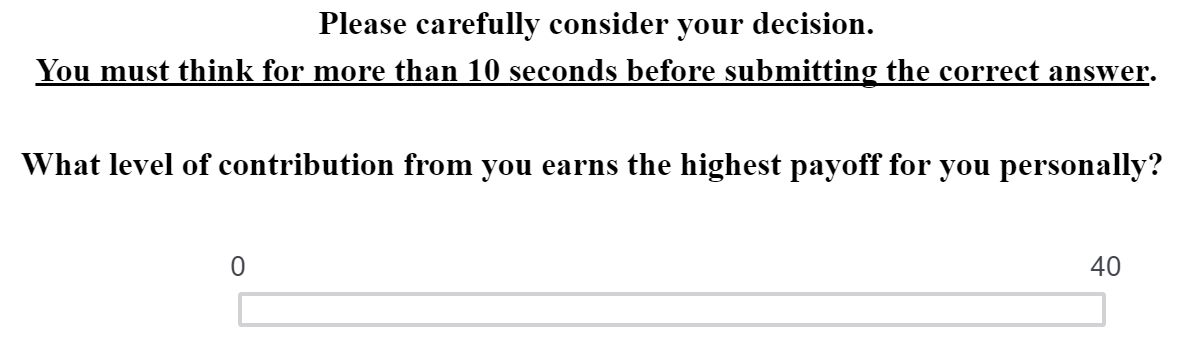


**References**

1. Bouwmeester S, Verkoeijen PP, Aczel B, Barbosa F, Bègue L, Brañas-Garza P, et al. Registered Replication Report: Rand, Greene, and Nowak (2012). Perspectives on Psychological Science. 2017 May;12(3):527-42. doi:10.1177/1745691617693624
2. Cokely ET, Galesic M, Schulz E, Ghazal S, Garcia-Retamero R. Measuring risk literacy: The Berlin numeracy test. Judgment and Decision Making. 2012 Jan 1;7(1):25.
3. Maule AJ, Hockey GR, Bdzola L. Effects of time-pressure on decision-making under uncertainty: changes in affective state and information processing strategy. Acta psychologica. 2000 Jun 30;104(3):283-301.
4. Rand DG, Greene JD, Nowak MA. Spontaneous giving and calculated greed. Nature. 2012 Sep 20;489(7416):427-30. doi:10.1038/nature11467
5. Watson D, Tellegen A. Toward a consensual structure of mood. Psychological bulletin. 1985 Sep;98(2):219-235.
6. Lerner JS, Li Y, Valdesolo P, Kassam KS. Emotion and decision making. Annual Review of Psychology. 2015 Jan 5;66:799-823.
